# Atypical Case of Posterior Reversible Encephalopathy in a Pregnant Patient Without Preeclampsia

**DOI:** 10.7759/cureus.5620

**Published:** 2019-09-10

**Authors:** Marvi Qureshi, Jeff Huang

**Affiliations:** 1 Medicine, University of Central Florida College of Medicine, Orlando, USA; 2 Anesthesiology, University of Central Florida College of Medicine, Orlando, USA

**Keywords:** posterior reversible encephalopathy, preeclampsia, seizure, pregnancy complications, spinal epidural anesthesia, spinal epidural anesthesia, eclampsia, hypertension, obstetrics and gynecology, emergency

## Abstract

We report a 33-year-old gravida one patient at 41 weeks gestation who had been admitted to the Labor and Delivery floor amid labor with seizures and no prior history of eclampsia, hypertension, or seizures. The patient was transported for an emergency cesarean section under general anesthesia. The patient’s epidural placed prior to the seizure was discontinued. The patient was extubated post-delivery. Neurology was consulted to determine the cause of first-time seizures. Computed tomography and magnetic resonance imaging revealed posterior reversible encephalopathy syndrome. Obstetricians and anesthesiologists should consider posterior reversible encephalopathy syndrome when performing Magnetic Resonance Imaging in previously healthy patients who initially present with seizures during labor, especially in patients who do not have hypertension.

## Introduction

One rare potential obstetrical emergency is posterior reversible encephalopathy syndrome (PRES), which may manifest itself in a variety of ways [1]. PRES is a neurological disorder characterized by vasogenic edema in the parietal and occipital lobes; it usually presents during parturition, which is when its symptoms typically manifest [2]. The most prevalent and early symptoms include seizures, headaches, encephalopathy, visual disturbances, and coma [1]. Common causes of this syndrome include pre-eclampsia and eclampsia, but patients who are initially normotensive and do not have proteinuria may also present with PRES, as in our patient [2]. We present the case of a 33-year-old gravida one (G1) patient with no prior history of seizures or hypertension who presented with seizures an hour into labor and was diagnosed with posterior reversible encephalopathy post-delivery.

## Case presentation

A 33-year-old Gravida (G) one patient at 41 weeks gestation presented for a scheduled induction of labor using misoprostol to avoid post-term pregnancy complications. Her history was positive for asthma, which was stable throughout the pregnancy. The patient did not report any prior history of neurological issues or allergies. Her medications were prenatal vitamins and Metamucil. On physical examination, she exhibited the normal physical changes of pregnancy. Her prenatal laboratory values were unremarkable. Her vital signs were within normal limits, with a blood pressure of 130/98, heart rate (HR) of 22 and a temperature of 98.6° Fahrenheit. She was 5 feet 4 inches tall and weighed 88.4 kg.

The patient requested an epidural for labor pain and was assessed by the anesthesia team before placement of the epidural catheter. Standard monitors were placed. Subsequently, an epidural catheter was placed without any complications. There was no cerebrospinal fluid or heme aspiration with the utilization of a 3 mL syringe. There was also a negative test dose. The patient was given 0.1% ropivacaine with 2 mcg/mL fentanyl at 10 mL/hour basal infusion with a demand bolus dose of 10 mL every 20 minutes. She was comfortable with the epidural analgesia, and the obstetrician reported no neurological symptoms. 

An hour into labor, the patient had an episode of seizures. The labor nurse reported that seizure activity started while she was supine in bed and the seizure appeared to be a generalized motor seizure. The patient had not had any changes in vital signs just before the seizure, while maintaining spontaneous ventilation throughout the seizure previously, with oxygen saturation (SpO2) of 95-100%. She was placed in the right lateral decubitus position on the bed, and supplemental oxygen was given by face mask. The patient-controlled epidural analgesia (PCEA) was turned off. Midazolam 2 mg IV was given, which caused her seizure to end. Thereafter, the patient was minimally responsive, in a postictal like state. The patient did not respond to questions but was awake with pupils responsive for approximately five minutes. After this, the patient was alert and oriented to person, place, and time, and did not recall the seizure. The patient was afebrile with blood pressure at 160/100 mmHg, tachycardia to the 140s and oxygen saturation above 95%, and with Glasgow Coma Scale at 15 without any neurological deficit. The patient did not experience any signs of fever at this time. Otherwise, the physical exam was normal. 

Before the seizure, fetal heart tones were noted to be in the 80s to 90s. During the seizure, the fetal heart tones increased to 130, then further increasing to 170s and 180s after the seizure. Due to the fetal heart tones, the surgeon proceeded with the cesarean section.

The patient was taken to the operating room for an emergency cesarean section under general anesthesia. The maternal vital signs remained relatively stable throughout the operation, as per her baseline before the seizure. Cesarean section proceeded without event. Induction of anesthesia was done with Propofol and succinylcholine intravenous and the patient was intubated. Anesthesia was maintained with FiO2 100% and sevoflurane 2%. A viable female infant was delivered weighing 3,710 g; Appearance, Pulse, Grimace, Activity, Respiration (APGAR) scores of one and seven and cord pH of 6.98. The patient received a total of 1,000 mL of intravenous fluids and had an estimated blood loss of 700 mL and urine output of 250 mL.

There was a grossly normal appearance of the uterus, ovaries, and tubes. There was no evidence of abruption of the placenta. There was no evidence of intra-amniotic infection.

The patient met criteria for extubation, she was extubated in the operating room and was transferred to the intensive care unit (ICU) because of new-onset seizure activity of unknown etiology and continued tachycardia in the 120s after delivery.

In the ICU, the patient denied any chest pain, palpitations, shortness of breath, vomiting or diarrhea, however she complained of 3/10 headache that began after delivery. Glasgow Coma Scale was 15. No acute neurologic deficits found on physical exam. The patient was started on seizure prophylaxis with Keppra and Ativan as needed. Her laboratory results were within normal limits except D-dimer. Her hemoglobin (Hgb) was 12.9 g/dL and hematocrit (Hct), 38.6%. Multidisciplinary teams were consulted including the radiologist, neurologist, and neurosurgery for the next three days. CT head showed a small amount of subarachnoid hemorrhage. No definite parenchymal hemorrhage was shown.

**Figure 1:**
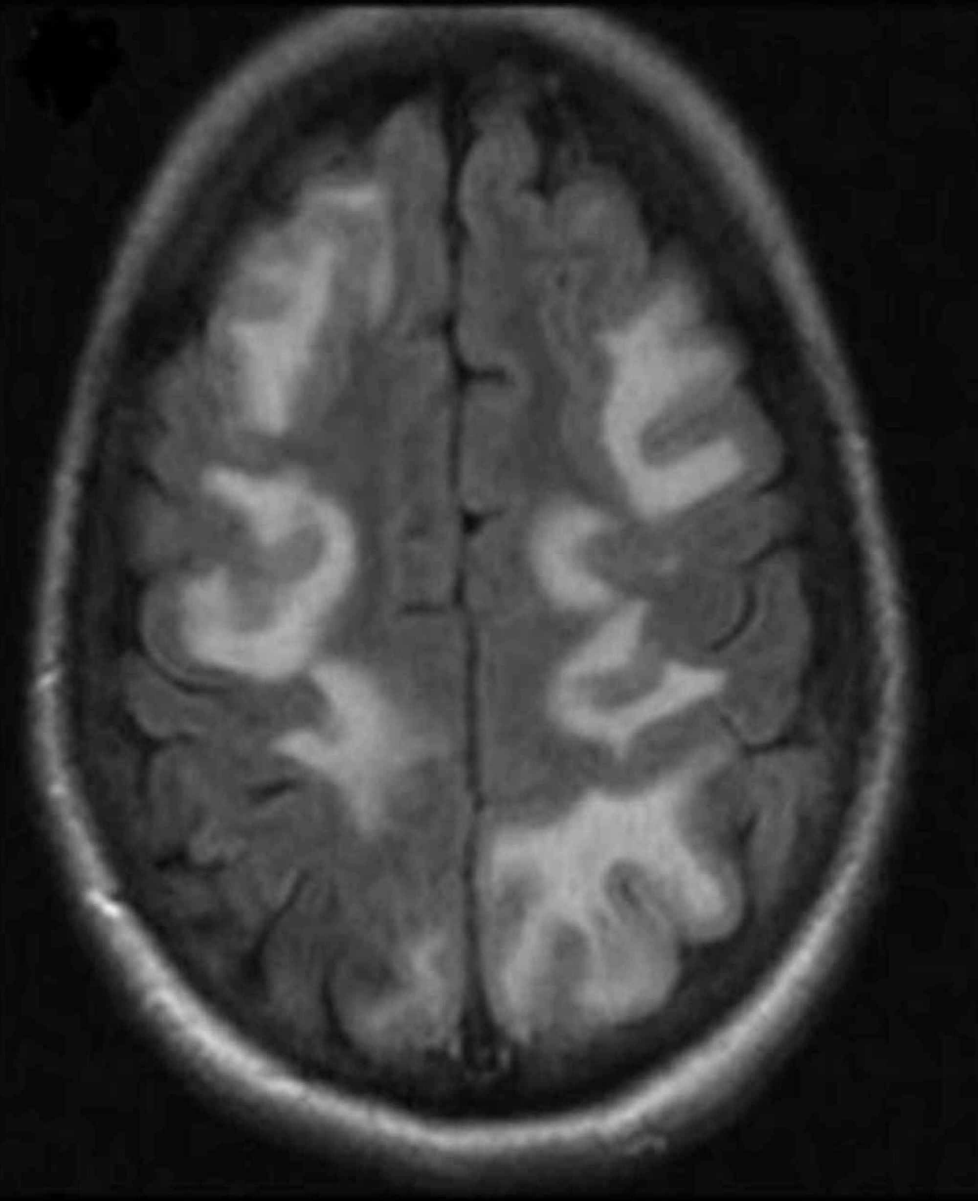
MRI findings of Posterior Reversible Encephalopathy Syndrome reproduced with permission from Wolters Kluwer Health Inc. License [3]

MRI of the brain was then obtained (Figure 1). It displayed extensive abnormal signal in the cortical grey matter, pons, and bilateral cerebral hemispheres. There was associated enhancement along the bilateral frontal lobes, at least some of which was cortically based. Differential considerations include an atypical presentation of posterior reversible encephalopathy syndrome or meningitis. There was no diffusion restriction to suggest infarct or hypoxic-ischemic injury. Bilateral findings would make status epilepticus unlikely. There was a small amount of extra-axial susceptibility artifact which is compatible with subarachnoid hemorrhage.

Magnetic resonance venography (MRV) of the head without contrast and magnetic resonance angiography (MRA) of the head with contrast were ordered to further establish the diagnosis. The MRI MRV was found to be normal with no evidence of vascular occlusion or thrombosis. The MRA head with contrast was normal, with no evidence of intracranial aneurysm or major vessel occlusion.

She had no further seizures during her stay.

Quantitative troponin levels were elevated at 0.06 ng/ml and further testing including echocardiogram and electrocardiogram were ordered. The echocardiogram displayed normal left ventricular size, thickness, systolic function, and wall motion. The visually estimated ejection fraction was between 60-64%. The electrocardiogram (EKG) showed normal sinus rhythm and was unremarkable.

The following day an electroencephalogram (EEG) was performed that was slightly abnormal due to a mild slowing of posterior dominant rhythm, diffuse theta slowing of the background mainly during drowsiness. The study did not capture any interictal epileptiform discharges or any electrographic ictal discharges.

The patient was discharged home after three days as her condition was stable with a normal neurological exam. Recommendations were to follow-up with neurology outpatient with the diagnosis of posterior reversible encephalopathy syndrome and repeat MRI in two months. The patient was continued on Keppra 500 mg twice a day.

## Discussion

Posterior reversible encephalopathy syndrome (PRES) is a syndrome that can present with symptoms such as seizures, headaches, visual disturbances, visual loss, as well as coma [1]. PRES has been shown to have an association with pre-eclampsia and eclampsia in pregnant patients but has been seen in patients without a formal diagnosis of either, as was the case in our patient [2]. Studies have been conducted to test the hypothesis that patients with preeclampsia and eclampsia who eventually go on to develop PRES likely share a pathophysiologic background. As such, a study conducted by Mayama et al. suggested magnetic resonance imaging studies in patients with preeclampsia and eclampsia to rule out PRES [4]. This is particularly important because various other studies have determined that pregnancy-related PRES may present with neuroimaging findings that are atypical. In a study conducted by Wen et al., nearly all imaged patients that had been diagnosed with eclampsia, in reality, had clinical and radiologic findings of PRES. This demonstrates the blurry line between PRES and eclampsia as well as preeclampsia [5]. The reasoning for the shared pathophysiology is not completely understood but it is important to consider. MRI testing should be considered more often, even in patients with a diagnosis of preeclampsia or eclampsia [5].

In addition, PRES has been found to be associated with organ transplantation, blood transfusion, infection, renal failure, hypertensive crises, chemotherapeutic agents, immunosuppressants, as well as autoimmune diseases. It is believed that this syndrome initially takes place due to vasogenic edema that is present in the parieto-occipital white matter, but there can also be edema that is present in the frontal lobes (such as in our patient), brainstem, or cerebellum [1]. The exact pathophysiology has been proposed to involve two different theories [1]. The first theory involves cerebrovascular autoregulation that is not properly regulated, resulting in increased perfusion that eventually destroys the blood-brain barrier [1,6]. Such breakdown results in the vasogenic edema that is typical of PRES [1]. The second theory involves vasoconstriction of the arterioles that leads to decreased blood flow, ischemia of related brain structures, and subsequent cytotoxic edema [1,7].

Atypical PRES involves areas including the frontal lobe, temporal lobe, cerebellum, or brainstem [1]. As such, our patient would be classified as atypical presentation. As described by its name, PRES is reversible and if diagnosed promptly, further consequences can be prevented [1]. The diagnosis is made through neuroradiologic findings as well as clinical presentations, focusing specifically on the characteristic findings on MRI of parieto-occipital gyriform lesions. Currently, brain MRI is the cornerstone for diagnosis because it aids in ruling out other potential diagnoses [8]. Clinical presentations of PRES include seizures, status epilepticus, headache, visual disturbances, but in many cases, the only clinical presentation may be headaches [9]. In addition, the majority of patients typically present with severe hypertension, but cases have been documented of patients with only mild hypertension or even normotensive patients, which could be the case in our patient [10]. Since our patient presented with these findings on MRI, as well as the clinical presentation of seizures, we were able to diagnose our patient with posterior reversible encephalopathy syndrome.

As shown in an analysis of 12 cases of PRES, if appropriate treatments were given, clinical and MRI follow-up showed a reversal of symptoms and radiological abnormalities [11]. Treatment is focused on correcting increased blood pressure, if present, and treating seizures [8]. The seizures are treated with magnesium sulfate, which is the drug of choice [8]. In patients who have received appropriate treatment, radiological testing has revealed normal findings after a year of treatment [8].

In a patient presenting with seizures during pregnancy, it would be important to consider other diagnoses and conditions such as underlying disease, eclampsia, or local toxicity before completely establishing a diagnosis of posterior reversible encephalopathy. Other relevant diagnoses that must be considered in a patient presenting with seizures during pregnancy include cerebral venous thrombosis, intracerebral hemorrhage, brain tumors, idiopathic epilepsy, infection (meningoencephalitis), amniotic fluid embolism, angiopathy, as well as metabolic disorders such as uremia, hypoglycemia, and hyponatremia [8]. When studying the various etiologies of status epilepticus during pregnancy, Rajiv and Radhakrishnan found that out of 15 patients who presented with status epilepticus, the etiology for six were due to PRES, four were due to eclampsia, three were due to cortical venous thrombosis, one was due to mesial temporal sclerosis, and one was due to occipital gliosis [11]. This study shows that PRES is not as uncommon as previously believed.

Any patient who presents with seizures during pregnancy should first be considered for eclampsia before other diagnoses, including PRES [11]. However, it was unlikely that our patient’s presentation was due to eclampsia because of the normal blood pressure prior to beginning labor and normal laboratory tests. However, Rajiv and Radhakrishnan discovered two patients for whom the etiology had previously been determined to be eclampsia had PRES-like changes observed on MRI when they had hypotension prior to the development of hypertension [11]. As such, eclampsia should have been considered in our patient as well, but since she did not progress to have hypertension, it was still not the leading diagnosis.

Other potential diagnoses for our patient included neoplasm, head trauma, angiopathy, cortical venous thrombosis, mesial temporal sclerosis, intracerebral hemorrhage, and occipital gliosis, all of which are diagnosed through imaging [11]. Blood culture, and the history and physical exam did not support infection. Considering the normal laboratory values for this patient, metabolic disorders were also ruled out as a likely cause for seizures in our patient.

Although our patient does not have a history of epilepsy, it is still important to consider a diagnosis of epilepsy in a pregnant patient with seizures. With the MRI displaying no diffusion restriction to suggest infarct or hypoxic-ischemic injury, the EEG confirmed that this patient did not have epilepsy. Bilateral findings would make status epilepticus unlikely. Epilepsy is an important cause of seizures that can take place during pregnancy [12]. Seizure prevention may require the use of medications throughout pregnancy, however, such prevention comes at a cost of teratogenic effects [8]. Such medications include valproate, carbamazepine, and lamotrigine [12]. As such, it is important to weigh the risks versus benefits of seizures to the teratogenic effect on the fetus [12]. Another important diagnosis to consider is local anesthetic systemic toxicity (LAST). LAST involves utilization of local anesthetic drugs that result in a life-threatening adverse effect [13]. Pregnancy has been found to increase the sensitivity of the patient to local anesthetics utilized during the delivery process, which leads to an increased risk of LAST [13]. Such toxicity usually takes place immediately following or up to an hour after the injection of the local anesthesia. LAST wasn’t present in our patient who had an epidural catheter placement for many hours prior to seizing. 

Central nervous system (CNS) toxicity, that manifests as seizures, is the most common presentation of LAST [14]. However, these seizures are also accompanied by other symptoms such as audio-visual disturbances, dysgeusia, and peri-oral paresthesia, none of which were seen in our patient [14]. These CNS findings are then found to progress to cardiovascular symptoms such as hypotension, conduction deficits, or dysrhythmias [14]. Again, such findings were not evident in our patient. In addition, a low concentration of local anesthetic was utilized. The cesarean section was also done under general anesthesia rather than utilize the epidural catheter to avoid local toxicity. Considering all of these factors and clinical presentation typically associated with patients who are presenting with LAST, the diagnosis is likely not LAST. 

## Conclusions

Our research indicates there is no report of anesthesia considerations related to an atypical case involving PRES in a normotensive pregnant patient. PRES should be considered as a diagnosis in a healthy pregnant woman who has a sudden onset of seizures during labor. Even from an anesthesia standpoint, as well as from a multidisciplinary approach, this diagnosis should be kept in mind because it can be confused with other diagnoses. The exact etiology of PRES during pregnancy is not known and remains controversial. Clinical presentation and MRI are used for diagnosis, and with prompt treatment of seizures, the syndrome is reversible. From an anesthesia perspective, for a patient who presents with symptoms similar to or characteristic of PRES, we should stabilize the patient, control the seizure, rule out LAST, maintain close communication with the obstetrician and other team members, develop a plan of care between the surgeon and anesthesiologist, and ultimately, ensure the safety of the mother and baby. 
